# Booster Vaccination: The Role of Reduced Antigen Content Vaccines as a Preschool Booster

**DOI:** 10.1155/2014/541319

**Published:** 2014-02-11

**Authors:** Giovanni Gabutti, Cecilia Trucchi, Michele Conversano, Giambattista Zivelonghi, Giorgio Zoppi

**Affiliations:** ^1^Department of Prevention, O.U. Hygiene and Public Health, LHU 4 “Chiavarese”-Liguria Region, Corso Dante 163, Chiavari, 16043 Genoa, Italy; ^2^Department of Health Sciences, University of Genova, Via Pastore 1, 16132 Genoa, Italy; ^3^Department of Prevention, LHU Taranto, Viale Virgilio 31, 74100 Taranto, Italy; ^4^Department of Prevention, LHU 20 Verona, Via Salvo d'Acquisto 7, 37122 Verona, Italy

## Abstract

The need for boosters for tetanus, diphtheria, pertussis, and polio, starting from preschool age, is related to the waning immune protection conferred by vaccination, the elimination/reduction of natural boosters due to large-scale immunization programs, and the possibility of reintroduction of wild agents from endemic areas. Taking into account the relevance of safety/tolerability in the compliance with vaccination among the population, it have been assessed whether today enough scientific evidences are available to support the use of dTap-IPV booster in preschool age. The review of the literature was conducted using the PubMed search engine. A total of 41 works has been selected; besides, the documentation produced by the World Health Organization, the European Centre for Disease Control, and the Italian Ministry of Health has been consulted. Many recent papers confirm the opportunity to use a low antigenic dose vaccine starting from 4 to 6 years of age. There is also evidence that 10 years after immunization the rate of seroprotected subjects against diphtheria does not differ significantly between those vaccinated with paediatric dose (DTaP) or reduced dose (dTaP or dTap) product. The dTpa vaccine is highly immunogenic for diphtheria toxoids regardless of prior vaccination history (2 + 1 and 3 + 1 schedules).

## 1. Introduction

The modern approach in vaccinology implies that the potential of newly available products is fully exploited by ensuring their rational use based on evidence-based epidemiology and focused on the objectives to be achieved. The vaccination schedule, indicating the chronological sequence of immunizations, is the essential tool for achieving the objectives of different vaccinations. New scientific evidences, organizational needs, and/or the availability of new vaccines imply the continuous updating of the calendar [1, 2]. The Italian vaccination schedule provides two primary doses of hexavalent vaccine (D, T, aP, HBV, Hib, IPV) with a booster at one year of age [3]. This schedule, commonly referred to by the acronym 2 + 1, was conceived in Italy in the 1970s and officially introduced in 1981 [4]. In several European countries is used, to date, the so-called 3 + 1 schedule, which includes a 3-dose primary series plus a booster in the second year of life. In both calendars (3 + 1 and 2 + 1) a booster dose of DTaP-IPV is then provided in preschool and, in many countries, even in adolescents [5]. The need for boosters for tetanus, diphtheria, pertussis, and polio [6], starting from preschool age, is related to the waning immune protection conferred by vaccination, the elimination or reduction of natural boosters due to large-scale immunization programs, and the possibility of reintroduction of wild agents from endemic areas.

In addition to these scientific and technological requirements, the opportunity to include another booster in the calendar is also linked to the alleged compliance that the population will have with this dose. In this context, the tolerability of the vaccines offered and the availability of combined vaccines, which permit reducing the number of injections, play a major role in achieving high vaccination coverage. Since many years, the administration in preschool age of the vaccine with a reduced antigen content (dTap-IPV) compared to the full dose one (DTaP-IPV) has showed a lower incidence and severity of local reactions, guaranteeing the same level of immunogenicity. The physiopathology of above-mentioned local reactions has not yet been fully elucidated. Several hypotheses have been formulated, taking into account the antigenic composition of vaccines (diphtheria and pertussis toxoids), high prevaccination antibody titers against diphtheria, tetanus and pertussis toxoids, impurities resulting from the production of toxoids, the use of aluminium as an adjuvant, and Th2-mediated cytokine production [7–15]. Such considerations would favour the introduction of dTap-IPV in the preschool age [16], but the same has not yet been recommended at European and national level [3, 5, 17]. In particular, fears have been expressed on the lower antibody titer elicited against diphtheria by dTap-IPV compared to DTaP-IPV and on the related shorter immune protection that this would allow [5, 18]. Taking into account the relevance of safety/tolerability in the compliance with vaccination among the population, and, in the case of Italy, having to increase vaccine coverage up to 95%, as prescribed by PNPV 2012–2014, it has been considered appropriate to assess whether today enough scientific evidences are available to support the use of dTap-IPV booster in preschool age.

## 2. Materials and Methods

The review of the literature was conducted using the PubMed search engine. The keywords used were as follows: “preschool,” “children,” “DTaP,” “DTaP-IPV,” “dTap,” “dTap-IPV,” and “adverse event.” The chosen keywords have been connected by the Boolean operator “and.” The limits applied to the research refer to the language used in publications (English or Italian). A total of 42 works have been selected; besides, the documentation produced by the technical bodies of the World Health Organization (WHO), the European Centre for Disease Control (ECDC), and the Italian Ministry of Health and published on their corporate websites has been consulted.

## 3. Results

### 3.1. Tolerability of Vaccines with Reduced Antigen Content When Used as Preschool Age Booster

The experts of the Global Pertussis Initiative (GPI), who met for the first time in 2002, had a favourable opinion to the use of the adult formulation (dTap-IPV) for preschool age booster. The analysis of the available studies indeed revealed that the immunogenicity elicited by the reduced antigen content vaccine is adequate and that tolerability is significantly higher than the full antigen content product [19]. In 2005, Huang et al. evaluated 60 Japanese children (age range: 6–8 years) who had been administered a dTap vaccine as a booster after the primary vaccination received in the first two years of life. Local reactions were reported more frequently than systemic symptoms; however, none of them was classified as grade 3 (i.e., symptoms that impair normal daily activities). Neither fever nor any serious adverse events have been reported. Given the higher reactogenicity of DTaP vaccine when administered as a booster with respect to its use in the primary cycle, the authors consider the reduced antigen content vaccine a viable alternative to the full antigen content vaccine, because it stimulates an adequate immune response and has better tolerability [20]. In 2006, Jacquet et al. have published a review of the available clinical trials regarding the use of DTaP-IPV in 5 countries as a fourth or fifth dose in preschool children or adolescents. The Italian and Swedish experience indicates that administration of this vaccine in 210 subjects aged 4 to 6 years has resulted in the onset of swelling, redness and pain at the site of inoculation, respectively, in 53.3%, 61%, and 71.4% of cases [21]. The values observed in this study, especially as regards local redness, have been higher than those after the use of the vaccine with reduced antigen content reported in the paper by Zepp and co-workers [22], cited by the authors. In a study published in 2007 and conducted in Germany, Sänger et al. have evaluated the immunogenicity and reactogenicity of three batches of a tetravalent dTap-IPV vaccine and of a dTap vaccine coadministered with a monovalent IPV, in subjects aged 4 to 8 years who had previously received a primary series with four doses of combined vaccines containing diphtheria-tetanus-pertussis components at full dose (DTaP). The pain at the injection site has been the most frequently reported symptom in both treatment groups. Local reactions have been reported less frequently in the dTap-IPV group than in those who have received dTap and IPV separately. Extended local reactions, involving the whole arm, were less frequent than those that would be expected with the use of the DTaP vaccine. In conclusion, the authors argue that, based on available data, the use of the vaccine with reduced antigen content may confer substantial benefits in terms of reactogenicity when used as a booster in preschool children, while ensuring adequate levels of immunogenicity [23]. In 2007 Bose et al. published a study, conducted in India in 2004, evaluating the reactogenicity and the acceptability of a dTap vaccine in 345 children in preschool age. The pain at the injection site was the most frequently reported symptom; a reaction of high grade, with impairment of normal daily activities, however transient and spontaneously resolved within 48 hours, has been reported in only 1.4% of cases. No one had reported extended swelling or redness. The authors argue that the reduced compliance with boosters in preschool children and adolescents could be due to the reactogenicity of full antigen content vaccines and that the use of a combined dTap vaccine could contribute to the acceptability of vaccination and to an increasing vaccination coverage, while ensuring adequate antibody responses [24]. A study published in USA in 2008 provided further information about immunogenicity and safety of DTaP-IPV compared with the coadministration of DTaP and IPV vaccines. The 4,209 study participants (age range: 4–6 years) had carried out a 3 + 1 series of DTaP in the first two years of life. Parents or legal representatives were asked to report local reactions that occurred during the 4 days following vaccination; the “Brighton Collaboration” classification [25] was used by measuring the circumference of the arm and the occurrence of systemic symptoms. The intensity of the symptoms was graded by a scale from zero to three. The grade 3 was referring to the redness and swelling with a diameter ≥50 mm. The staff participating in the study had to be contacted by parents and legal representatives in cases of swelling of grade 3, or anyway interfering with daily activities, and an increase in arm circumference >30 mm had occurred. Local pain was the most frequently reported symptom by both treatment groups. Extended swelling manifested frequently and with moderate to intense grade in most cases. These results are in line with other studies carried out in the same age group [26]. In 2008, Meyer et al. published the results of a clinical trial conducted in Germany comparing immunogenicity and reactogenicity of vaccines with full and reduced antigen content administered to children in preschool age (from 4 to 6 years of age). The dTap vaccine has demonstrated better tolerability than the vaccine with paediatric dosage commonly used in this age group. In particular, the incidence of local reactions was higher in the group that received the DTaP vaccine compared to the group that received the vaccine with reduced antigen content. Although local reactions such as swelling at the site of inoculation do not imply long-term clinical consequences, the authors argue that this aspect can have an impact on the compliance with boosters by parents [27]. A Japanese study, published in 2008 by Lin et al., involved 85 children between 6 and 7 years of age and had the aim of evaluating the immunogenicity and reactogenicity of dTap-IPV. Reactogenicity data showed that the vaccine is well tolerated and that no serious adverse event occurred. The pain at the injection site was the most frequently reported symptom. No subjects reported local swelling extended in the 48 hours following the administration of the vaccine. The authors conclude that the use of the reduced antigen content vaccine provides a useful tool to reduce the significant reactogenicity observed in other studies following repeated administration of full dose vaccines [28]. The results of two studies conducted in China on 690 children aged between 6 and 8 years to evaluate the immunogenicity, reactogenicity, and safety of a dTap vaccine were published in 2010. The authors conclude that the vaccine is highly tolerated and immunogenic and are therefore in favour of the use of reduced dose vaccine as a booster in preschool age [29]. A study, conducted in Australia by Quinn et al. and published in 2011, involved 54 subjects who developed an extensive local reaction following the administration of the fourth dose of DTaP vaccine in the second year of life. The main objective of the study was to compare the rate of local reactions following the use of full antigen dose or reduced antigen content vaccines as booster in preschool age. The majority of subjects (72%) developed local reactions at the injection site and, in particular, almost half (46.2%) reported large swelling. This type of reaction was significantly less frequent in the group of subjects who had received the dTap vaccine than the DTaP one (25% versus 60.9%). The authors, even taking into account the small size of the sample studied, found clear lower reactogenicity of the dTap vaccine than the vaccine at full dose [30]. In 2012 a study was published, conducted in Thailand by Pancharoen et al., evaluating immunogenicity and safety of a DTaP-IPV vaccine administered as a booster in preschool age (4–6 years). Eighty-seven percent out of 123 vaccinated subjects reported at least one adverse event. In particular, 82.1% of participants experienced local reactions, and, more specifically pain (75.6%), redness (48.8%), and swelling (36.6%). The authors have stressed the importance of the use of combined vaccines in order to obtain the best results in terms of vaccine coverage and have also observed that local adverse reactions increase with age and the number of booster doses administered [31]. At the 30th ESPID Congress, which took place in Greece in 2012, Alguacil Ramos et al. presented the results of a retrospective analysis, conducted in Spain between 2009 and 2011, on adverse events following the administration of DTaP and dTap vaccines in preschool children. This study involved 98,398 children vaccinated with the fifth dose of diphtheria, tetanus, and whooping cough at the age of 5-6 years, as provided by the Spanish vaccination schedule. The rate of adverse events reported through the national vaccine-vigilance network was 1.11‰ in the DTaP group and 0.52‰ in the dTap group. In particular, local swelling was the most frequently reported adverse event following the DTaP vaccine (29.6% of cases). The authors, even if noting a lower incidence of adverse events in subjects who received the dTap vaccine, pointed out that all reactions reported were of low grade [32]. Ferrera et al. in 2012, at last, evaluated immunogenicity, safety, and reactogenicity of a booster dose of dTap-IPV vaccine compared with a full-dose DTPa-IPV vaccine in Italian children aged between 5 and 6 years. The analysis involved 151 subjects vaccinated with dTap-IPV and 152 subjects immunized with DTaP-IPV. During the follow-up period of 4 days participants were asked to record and describe the occurrence of local and systemic adverse events. Pain and redness at the injection site have been reported less frequently by subjects who received the vaccine with reduced antigen content. In detail, local pain was the most frequently reported local symptom by both treatment groups (dTap-IPV 58.9%, DTaP-IPV 61.2%, *P* = 0.69). These data demonstrate that the vaccine with reduced antigen content is related to a lower incidence of local reactions than the vaccine with paediatric dose. The authors believe that the lower reactogenicity of reduced antigen content formulations can be an advantage in order to maintain adequate vaccine coverage rates in preschool and adolescent age [33].

The results of all these studies are summarized in Tables 1 and 2.

### 3.2. Immunogenicity of Vaccines with Reduced Antigen Content When Used as Preschool Age Booster

Generally the immunogenicity of dTap-IPV vaccines is compared with the one of DTaP-IPV vaccines or equivalent combinations by measuring antibody concentrations and evaluating seroprotection markers, if available (e.g., tetanus or diphtheria), or proxy values of protection (e.g., pertussis). Products with different antigen content do not differ greatly from each other in terms of immunogenicity as far as T, aP, and IPV antigens are considered. Taking into account the antibody production against diphtheria antigen, the antibody amount elicited by paediatric (full dose) vaccine is higher than the one obtained with the reduced content antigen vaccine. A study conducted in Italy by comparing the two formulations showed a statistically higher geometric title against diphtheria (anti-D) in subjects who had received DT than that observed in subjects treated with dT (14.1 IU/mL versus 7.7 IU/mL, *P* < 0.001). The authors consider that the higher antibody response and the comparable reactogenicity of the two products would suggest a preferential use of the DT product in preschool age, especially if no additional boosters in adolescence or adulthood are provided [18]. It is noteworthy that data in this paper show that the use of standard dose (DT) resulted in a significantly higher frequency of redness, swelling, and pain at the injection site, of any degree or severity. Besides, the vaccines used in this study did not include the dTap vaccine currently in use in Italy, as it was not yet commercially available. The reduced antigen content product currently available has a diphtheria antigen content of 2.5 Lf and thus is greater than the vaccines evaluated in this Italian study (2 Lf of diphtheria toxoid). With regard to the duration of protection, a study that evaluated, using a mathematical model, the diphtheria seroprotection rates achieved in adults and children (5-6 years of age) 10 years after the use of dTap or DTaP vaccines was published in 2004. In detail, the model took into account the antibody titers measured in subjects immunized with DTaP or dTap up to 3.5 years after administration of vaccines and assessed the antibody decay in 10 years. The results have shown that the expected seroprotection 10 years after the administration of DTaP or dTap is substantially comparable. The authors believe that it is unlikely that the minimum difference in diphtheria antibody concentration found between the two vaccines could be of clinical relevance 10 years later at the time of the adolescent booster [34].

Following this type of evaluation, results of some field studies became available.

The aforementioned randomized trial conducted by Sänger et al. and published in 2007 was based on the administration of a dTap-IPV dose compared to the coadministration of a dTap and IPV dose in children aged 4–8 years previously immunized with 4 doses of DTaP. One month after the booster dose, the seroprotection rates and the immune responses were similar in both groups; at least 99.9% of subjects had protective titers against diphtheria, tetanus, and polio, and at least 90.1% had a response to the pertussis antigens. A year after the booster, 98.6% of the subjects continued to have protective titers against tetanus, diphtheria, and polio, and 81.2% were seropositive against the pertussis components. In this study, the safety and tolerability profile was satisfactory [23]. These data confirm what was previously obtained in researches conducted in Thailand and Taiwan [20, 35]. The previously mentioned study written by Meyer et al. and published in 2008 concerned children who had received the primary vaccination with four doses of DTaP and at the age of 5-6 years had received a booster dose of DTaP or dT or dTap. The evaluation of antibody titers was performed in serum samples collected one month before, one month after, and 3.5 years after the booster. The reduced antigen content vaccine induced an effective booster response to all antigens contained in it when given to children in preschool age; this response was evaluated in terms of both humoral and cell mediated response. It is noteworthy that 1 month after the booster dose no significant differences in terms of seroprotection against tetanus and diphtheria and of response to pertussis antigens were observed. After 3.5 years, seroprotection levels were similar and antibody titers had declined with a similar trend. Furthermore, the use of reduced antigen content vaccine was related to a better tolerability profile than the paediatric (full dose) product. In conclusion, the study showed that both the paediatric (full dose) and the reduced antigen content vaccine elicit a similar level of long-term seroprotection against antigens contained in them [27]. In 2009, McIntyre et al. evaluated the seroprotection against diphtheria and tetanus in adults (mean age: 45.6 years) after the administration of a dose of dTap or the coadministration of monovalent acellular pertussis vaccine and dT. The study showed that 5 years after booster vaccination the rate of subjects with seroprotective levels against tetanus and diphtheria as well as geometric mean titers was higher than those detected before the same vaccination. Seroprotection against diphtheria was present in 94.4% and 93.7% of those who had received a booster with dTap or dT, respectively (96.2% and 90.6% for tetanus) [36]. In 2009, Mertsola et al. reported the results of the assessment of the administration of booster at decennial intervals in people belonging to different age groups. One study showed that the repeated use of dTap in adults had a reactogenicity profile similar to that detected after repeated boosters with dT [37]. Another study evaluated immunogenicity and safety of a dTap booster administered after 6 or 10 years in adolescents (9–13 years) and young adults (20–24 years), showing the achievement of a significant immune response against tetanus and diphtheria, and a significant increase (8–15 times) of the geometric mean titers against pertussis antigens, with a good tolerability profile [38]. The assessment of the immunogenicity of repeated administrations of dTap vaccines in young adults (mean age: 21 years) and adults (mean age: 50 years) has allowed the identification of the kinetics of the geometric mean titers against the different antigens. Concerning diphtheria, a significant increase 1 month after the first booster, the maintenance of a protective level of antibodies in the following decade, and then a significant immune response after a new booster have been shown [39]. The results obtained in young adults were subsequently published in 2010, supporting the use of dTap vaccine as a decennial booster [40]. In 2010 Knuf et al. published the results of a study conducted in adolescents, previously vaccinated at the age of 4–8 years with dTap-IPV or dTap, in order to evaluate immunogenicity, safety, and reactogenicity of a dTap-IPV booster dose. All subjects had received previous vaccination during infancy with four doses of DTaP. A second dose of dTap-IPV in these adolescents was highly immunogenic and well tolerated, supporting the opportunity of repeated boosters. It is noteworthy that prior to administration of the dose at the age of 9–13 years, at least 97% of subjects who had received a dose of dTap-IPV five years before had protective antibody levels against diphtheria, tetanus, and polio. Concerning diphtheria, before the booster dose in adolescence, 89.2% and 85.5% of subjects were seroprotected by ELISA, respectively, having previously received dTap-IPV or dTap. By using the neutralization test, the seroprotection rate was equal to 98.2% and 95.2%, respectively, in those who had previously received dTap-IPV or dTap [41]. In 2011, Scott has published a review on the results obtained with a dTap vaccine containing 5 pertussis components. In clinical trials conducted in the UK and North America the use of a booster dose with this vaccine elicited a robust immune response against all antigenic components in children aged 4 years or more, in adolescents and adults, and the safety and tolerability profile of the tested vaccine was good [42].

The results of these studies are summarized in Table 3.

## 4. Discussion

The goal of keeping the immune protection conferred by childhood vaccinations high has been addressed in the National Vaccine Plan 2005–2007 that highlighted the existence of indications for the use of booster vaccinations for tetanus, diphtheria, pertussis, and even polio, starting from preschool age [17]. In particular, the document identified two times of action: 5-6 years (for tetanus, diphtheria, pertussis, and polio) and 11–15 years (for tetanus, diphtheria, and pertussis). Subsequently, the possibility of a booster at the age of 5-6 years with reduced antigen content vaccine (dTap) was included in the immunization schedule by the Autonomous Province of Bolzano [43]. The calendar of the Apulia Region already in 2009 reported that in children 6 years of age the use of dTap formulation adults was indicated [44]. With regard to the 2 + 1 calendar, as most of the evidence on the use of the vaccine with reduced antigen content dTap (or dTap-IPV) in preschool age comes from experience in countries with a 3 + 1 schedule, it is relevant that the level of safety, tolerability, and immunogenicity for all antigenic components contained in the hexavalent vaccine is adequate regardless of the vaccination schedule adopted (3 + 1 or 2 + 1) in the first year of life [12, 23, 27, 33, 45–48].

In June 2010 a working group was set up with the aim of improving the practice of booster vaccination with diphtheria-tetanus-pertussis-polio in preschool age in Italy. Local experience brought by members of the group totalled tens of thousands of doses of dTpa/dTpa-IPV administered since 2003 and the general perception of tolerability by health care providers was “good” and better than the previous DTPa and “good” for the user. The preference for the dTap-IPV is based also on logistical aspects (one vaccine for all boosters). The conclusions of the Working Group indicate that the strategic choice of the use of dTap-IPV for the 4th dose is rational and well documented on the basis of the findings of published data and of the experience of health care personnel working in vaccination services [16]. In April 2012, a vaccination schedule (called “Roadmap for Life”) has been set up by the Italian Society of Hygiene, Preventive Medicine and Public Health (SItI), the Italian Federation of Paediatricians (FIMP), the Italian Society of Pediatrics (SIP), and the Italian Society of General Practitioners (SIMMG). The document specifies that it is possible to use the dTap vaccine starting from 4 years of age provided that high vaccine coverage in adolescence is guaranteed; besides, the need for dTap decennial boosters starting from adolescence is taken up and supported [49]. In 2011 an update of the recommendations on the use of dTap vaccine from the Advisory Committee on Immunization Practices (ACIP) was also published in the “Morbidity and Mortality Weekly Report.” The immune response elicited by the administration of a booster dose of vaccine containing a reduced antigen dose in preschool children was considered comparable to that obtained using the full dose vaccine; for these reasons, the ACIP supports the use of dTap vaccine in this age group [50]. In a review of the available studies on combined vaccines published in 2011, Skibinski et al. argue that DTaP vaccines cause extended local reactions, in particular local swelling, in a significant number of subjects, whose frequency and severity are related to the number of doses administered. The available data supporting the use of reduced antigen dose vaccine as a booster in preschool age are consistent and some countries, such as Germany and the UK, have already included in national calendars the administration of a booster dose of dTap or dTap-IPV in the age group 3–6 years [51].

## 5. Conclusions

The opportunity to use a low antigenic dose vaccine starting from 4 to 6 years of age is based on a number of recent papers that confirm the excellent safety and immunogenicity profile of these products. There is also evidence that, 10 years after immunization (internationally recommended time interval for booster vaccination), the rate of seroprotected subjects against diphtheria does not differ significantly between those vaccinated with paediatric dose (DTaP) or reduced dose (dTap) product. Considering the need to increase boosters starting from preschool age and that health professionals as well as users pay an increasing attention to safety and reactogenicity of vaccines, the availability of products with reduced antigen content, which ensure a high level of safety and immunogenicity and a lower reactogenicity compared to paediatric full dose vaccine, may contribute as early as preschool age to achieve and maintain high coverage rates.

## Figures and Tables

**Table 1:** Percentage of children reporting local symptoms during 4–15 days following booster vaccination.

Country and author	Age (years)	Vaccine	Local symptoms (%)										
			Pain		Redness		Swelling					Functional impairment	
			Any	Grade 3*	Any	Grade 3	Any	Grade 3****	Local	Diffuse	Adj. joint	Any	Grade 3
USA Black et al., 2008 [26]	4–6	DTaP − IPV	57.0	1.6	36.6	17.6**	26.0	10.2****	—	—	—	—	—
		DTaP + IPV	53.3	0.6	36.6	20.0**	27.0	11.5****	—	—	—	—	—

India Bose et al., 2007 [24]	4–6	dTap	31.1	1.4	8.9	—	18.2	—	—	—	—	—	—

Taiwan Huang et al., 2005 [20]	6–8	dTap	66.7	0.0	35.0	0.0**	40.0	0.0	—	—	—	—	—

Sweden, Italy Jacquet et al., 2006 [21]	4–7	DTaP − IPV	71.4	2.9	61.0	25.7**	53.3	13.3****	—	—	—	—	—

Taiwan Lin et al., 2008 [28]	6–8	dTap − IPV	37.6	9.4	28.2	0.0***	28.2	0.0***	—	—	—	—	—

Germany Meyer et al., 2008 [27]	4–6	dTap	47.8	1.1	33.9	13.3**	32.2	13.3****	—	—	—	—	—
		DTaP	63.3	2.2	48.9	23.3**	43.3	15.6****	—	—	—	—	—

Thailand Pancharoen et al., 2012 [31]	4–6	DTaP − IPV	75.6	0.8	48.8	4.1**	36.6	2.4****	—	—	—	—	—

Australia Quinn et al., 2011 [30]	4–6	DTaP	47.4	4.3	86.9	86.9**	69.5	60.8****	26.1	8.7	60.9	34.8	0.0
		dTap	50.1	0.0	100	93.8**	81.3	75.0****	56.3	18.8	25.0	18.8	0.0

Germany Sänger et al., 2007 [23]	4–8	dTap − IPV	55.7	2.8	53.2	10.6**	44.8	8.8****	9.6	2.7	0.4	—	—
		dTap − IPV	64.0	6.6	57.4	16.2**	54.4	14.0****	9.6	3.7	1.5	—	—

China Zhu et al., 2010 [29] Study A	6–8	dTap	10.0	—	23.0	10.0**	—	—	—	—	—	—	—

China Zhu et al., 2010 [29] Study B	6–8	dTap DT	19.0 20.5	— —	14.5 15.5	— —	12.0 8.0	— —	— —	— —	— —	— —	— —

*Cries when limb is moved/spontaneously painful.

**Redness with diameter >50 mm.

***Redness/swelling with diameter >20 mm.

****Swelling with diameter >50 mm, or noticeable diffuse swelling, or noticeable increase of limb circumference.

**Table 2:** Percentage of children reporting general symptoms during 4–15 days following booster vaccination.

Country and author	Age (years)	Vaccine	General symptoms (%)																			
			Fever		Drowsiness		Fatigue		Malaise		Myalgia		Loss of appetite		Irritability		Gastrointestinal (diarrhea)		Vomiting		Headache	
			≥37.5°C	>39.0°C	Any	Grade 3^*∧*^	Any	Grade 3^*∧*^	Any	Grade 3^*∧*^	Any	Grade 3^*∧*^	Any	Grade 3^*∧∧*^	Any	Grade 3^*∧*^	Any	Grade 3^*∧*^	Any	Grade 3^*∧*^	Any	Grade 3^*∧*^
USA Black et al., 2008 [26]	4–6	DTaP − IPV DTaP + IPV	16.0 14.8	1.1 1.1	19.1 17.5	0.8 0.8	16.7 —	0.0 —	— —	— —	— —	— —	15.5 16.0	0.8 0.6	— —	— —	6.7 —	0.0 —	— —	— —	— —	— —

India Bose et al., 2007 [24]	4–6	dTap	17.3	0.0	4.3	0.3	—	—	—	—	—	—	9.5	0.0	8.1	0.6	—	—	—	—	—	—

Taiwan Huang et al., 2005 [20]	6–8	dTap	0.0	0.0	—	—	16.7	0.0	—	—	—	—	—	—	—	—	6.7	0.0	—	—	10.0	0.0

Sweden, Italy Jacquet et al., 2006 [21]	4–7	DTaP − IPV	21.0	0.8	—	—	—	—	—	—	—	—	—	—	—	—	—	—	—	—	—	—

Taiwan Lin et al., 2008 [28]	6–8	dTap − IPV	11.8	—	10.6	0.0	—	—	—	—	—	—	12.9	0.0	5.9	—	—	—	—	—	—	—

Germany Meyer et al., 2008 [27]	4–6	dTap DTaP	20.0 22.2	5.6 4.4	— —	— —	— —	— —	— —	— —	— —	— —	17.8 17.8	0.6 1.1	16.1 21.1	0.6 2.2	10.0 8.9	0.0 0.0	7.8 4.4	0.6 0.0	— —	— —

Thailand Pancharoen et al., 2012 [31]	4–6	DTaP − IPV	10.6	0.8	—	—	—	—	32.5	0.0	43.9	0.0	—	—	—	—	—	—	—	—	24.4	0.0

Australia Quinn et al., 2011 [30]	4–6	DTaP dTap	17.4 6.3	— —	— —	— —	— —	— —	— —	— —	— —	— —	— —	— —	— —	— —	— —	— —	— —	— —	— —	— —

Germany Sänger et al., 2007 [23]	4–8	dTap − IPV dTap-IPV	22.1 13.2	6.0 1.5	20.7 22.8	2.6 0.0	— —	— —	— —	— —	— —	— —	18.9 17.6	2.9 0.0	17.5 16.2	0.6 0.0	— —	— —	— —	— —	— —	— —

China Zhu et al., 2010 Study A [29]	6–8	dTap	7.0	—	—	—	3.0	—	—	—	—	—	—	—	—	—	—	—	—	—	—	—

China Zhu et al., 2010 Study B [29]	6–8	dTap DT	17.0 13.5	— —	— —	— —	4.0 3.0	— —	— —	— —	— —	— —	— —	— —	— —	— —	3.0 4.0	— —	— —	— —	3.0 4.5	— —

^∧^Interfering with normal activities

^*∧∧*^Not eating at all

**Table 3:** Seroprotection rate (%) up to 10 years after booster vaccination of children.

Country and/or author	Age (years)	Vaccine	Timing	Seroprotection rate (%)							
				Anti-D	Anti-T	Antipolio type 1	Antipolio type 2	Antipolio type 3	PT	FHA	PRN
Cheuvart et al., 2004 [34]	4–6	dTap	After 1 month After 3.5 years	100 100	— —	— —	— —	— —	— —	— —	— —
		DTaP	After 1 month After 3.5 years	100 100	— —	— —	— —	— —	— —	— —	— —
		Td	After 1 month After 3.5 years	100 100	— —	— —	— —	— —	— —	— —	— —

Germany Sänger et al., 2007 [23]	4–8	dTap – IPV	After 1 month After 1 year	100 100	99.9 99.8	100 100	100 100	100 99.8	99.6 81.2	100 100	99.9 98.1
		dTap + IPV	After 1 month After 1 year	100 100	100 98.8	100 100	100 100	100 98.6	99.2 75.3	100 98.8	100 97.5

Thailand Kosuwon et al., 2003 [35]	4–6	dTap	After 1 month	99.4	100	—	—	—	96.9	96.9	95.1

Taiwan Huang et al., 2005 [20]	6–8	dTap	After 1 month	100	100	—	—	—	94.9	98.3	96.6

Germany Meyer et al., 2008 [27]	4–6	dTap	After 1 month After 3.5 years	100 100	100 98.4	— —	— —	— —	100 58.7	100 100	100 99.2
		DTaP	After 1 month After 3.5 years	100 100	100 98.5	— —	— —	— —	100 60.6	100 100	100 100
		dT	After 1 month After 3.5 years	100 100	100 100	— —	— —	— —	— —	— —	— —

Finland Mertsola et al., 2010 [40]	20–24	dTap	After 10 years	98.6	97.3	—	—	—	61.3	100	96.0
